# Traumatic Brain Injury and Firearm Use and Risk of Progressive Supranuclear Palsy Among Veterans

**DOI:** 10.3389/fneur.2018.00474

**Published:** 2018-06-20

**Authors:** Kristen D. Kelley, Harvey Checkoway, Deborah A. Hall, Stephen G. Reich, Chris Cunningham, Irene Litvan

**Affiliations:** ^1^School of Medicine, University of California, San Diego, La Jolla, CA, United States; ^2^Department of Family Medicine & Public Health, University of California, San Diego, La Jolla, CA, United States; ^3^Department of Neurology, Rush University Medical Center, Chicago, IL, United States; ^4^Department of Neurology, University of Maryland, Baltimore, MD, United States; ^5^Clinical Trials Unit, University of Louisville, Louisville, KY, United States; ^6^Department of Neurosciences, University of California, San Diego, La Jolla, CA, United States

**Keywords:** PSP, case-control study, military, firearms, traumatic brain injury

## Abstract

**Background:** Progressive supranuclear palsy (PSP) is a tauopathy that has a multifactorial etiology. Numerous studies that have investigated lead exposure and traumatic brain injury (TBI) as risk factors for other tauopathies, such as Alzheimer's disease, but not for PSP.

**Objective:** We sought to investigate the role of firearm usage, as a possible indicator of lead exposure, and TBI as risk factors for PSP in a population of military veterans.

**Methods:** We included participants from a larger case-control study who reported previous military service. Our sample included 67 PSP cases and 68 controls. Participants were administered a questionnaire to characterize firearm use in the military and occurrence of TBI.

**Results:** Cases were significantly less educated than controls. In unadjusted analyses, the proportion of PSP cases (80.6%) and controls (64.7%) who reported use of firearms as part of their military job was positively associated with PSP, odds ratio (OR) 2.2 (95% CI: 1–5.0). There were no significant case-control differences in mean service duration. There was only a weak association with history of TBI, OR 1.6 (95% CI: 0.8–3.4). In multivariate models, firearm usage (OR 3.7, 95% CI: 1.5, 9.8) remained significantly associated with PSP.

**Conclusions:** Our findings show a positive association between firearm usage and PSP and an inverse association between education and PSP. The former suggests a possible etiologic role of lead. Further studies are needed to confirm the potential etiologic effects of metals on PSP.

The study was registered in clinicaltrials.gov. ClinicalTrials.gov Identifier: NCT00431301.

## Introduction

Progressive supranuclear palsy (PSP) is a rare neurodegenerative movement disorder, and the most common of the atypical parkinsonian disorders. Pathologically, PSP is one of the tauopathies, a group of diseases in which tau, a protein involved in stabilizing microtubules, becomes hyperphosphorylated and forms insoluble aggregates in neurons (neurofibrillary tangles, NFTs) and astrocytes (tufted astrocytes) (1). The causes of PSP are poorly understood, although, the etiology is hypothesized to be multifactorial, evidenced by research indicating etiologic contributions from genetics, and various environmental exposures (2–5).

To our knowledge, neither heavy metal exposure nor traumatic brain injury (TBI) have been investigated as risk factors for PSP. However, many studies have investigated both heavy metal exposure and TBI as risk factors for other tauopathies, including Alzheimer's disease (AD) and chronic traumatic encephalopathy (CTE) (6–10). Due to the high prevalence of TBI and increased potential risk of heavy metal exposure due to use of firearms, military veterans are an appropriate population to investigate both of these potential risk factors.

It has been well-established that the use of firearms, in both indoor and outdoor ranges, increases exposure to lead and other heavy metals (11, 12). Occupational exposure studies have found increased blood lead levels in individuals who regularly use firearms as part of their jobs, including police officers and firearm instructors (13–15).

The relationship between lead exposure and cognitive decline has been well-established based on numerous reports from the Normative Aging Study (16, 17). These findings, among other, sparked interest in investigating a potential link between lead exposure and tauopathies which commonly present with cognitive decline (6–8, 18). One of the earliest mentions of the potential connection between lead exposure and tauopathy actual predates these studies and was a case report published in 1975. This report described the autopsy findings of an individual who died at age 42 due to worsening of lead encephalopathy. Post-mortem examination demonstrated a tenfold increase in lead in specific areas of the patient's brain compared to health controls. On histological examination there were numerous NFTs in addition to diffuse cortical atrophy which was thus attributed to the lead exposure (18). Since this early observation, several subsequent studies have investigated the potential connection between lead exposure and tau pathology (6–8). It has been shown that early exposure to lead can, in fact, alter the tau gene and gene expression (19). Additionally, in both rodent and primate models, early exposure to lead was reported to be associated with increased levels of total tau protein and tau mRNA, as well as increased levels of hyperphosphorylated tau and an increased presence of NFTs on brain tissue (7, 8). Rodent knock-out models have also confirmed that the tau pathway is required for lead to effect cognitive function (20).

Another risk factor for developing tauopathies is TBI. It has been well established that experiencing TBI increases the risk of developing AD (21–25). Much of the research establishing this link has been in the veteran population, due to the increased prevalence of TBI in these individuals (23, 24). Additionally, CTE, another tauopathy, is strongly linked to TBI. CTE was first described in professional boxers, but now is being recognized in other contact sport athletes, military veterans, and other civilians. Though initially CTE was mainly thought to be caused by more severe head injuries, there is now evidence that repetitive mild trauma can lead to this pathology (10).

In this study, we sought to investigate the role of these potential risk factors for PSP that may be more prevalent within the military population. Specifically, we aimed to explore the effect of the use of firearms, which typically causes lead exposure, and head injury on the development of PSP. Based on the current literature, we hypothesized that PSP cases will be more likely to report the use of firearms in the military and having experienced TBI with and without loss of consciousness.

## Materials and methods

### Participants

This study included all male participants from a larger multi-center case control study examining environmental and genetic risk factors associated with PSP (5) who reported having served in the US military. PSP patients (i.e., cases) were recruited from 15 sites across North America. Internal Review Board approval was obtained at each participating site. All participants completed a consent form for enrollment in this study. The original study cohort included 350 PSP participants, 284 matched cases and controls have been previously reported (5). Site PIs who recruited more than seven participants for the present study were invited to be included as authors, while those who recruited fewer participants are acknowledged.

As previously reported (5), PSP cases were incident cases, diagnosed within 1 year preceding the interview by the site principal investigator (PI). Diagnosis was based on the NINDS-SPSP criteria (26). Of the 67 PSP cases included in the present study, 87% met the NINDS-SPSP criteria for clinically probable or neuropathologically definite PSP. The remaining patients (*n* = 9) met criteria for clinically possible PSP. The majority of PSP cases (*n* = 53) had a Mini Mental Status Examination (MMSE) score ≥25 (27). Approximately 20% (*n* = 14) of the participants scored 24 or less on the MMSE, however, the primary site PI believed these participants did not meet criteria for major neurocognitive impairment and thus they were included.

PSP cases identified an age (±5 years) and gender matched non-blood related control, usually non-blood related in-laws. Identified controls were screened for cognitive impairment using the Telephone Interview for Cognitive Status (28) and for Parkinsonism using a telephone interview (29).

For each case, and their associated control, the reference year was defined as 10 years prior to the date of first reported PSP symptom. The purpose of this time frame was to account for the hypothesized lag time for PSP to manifest clinically. Though there is no published literature on the length of this lag time, we chose 10 years to be on the conservative side and avoid including possible preclinical and prodromal disease periods.

For the present study, we limited the analysis to cases and controls who reported having served in the military because of the greater prevalence of the exposures of interest. We additionally excluded women participants as there were only two women veterans in our sample. The final sample included 67 PSP cases and 68 controls.

### Measures

All participants were administered a modified telephone questionnaire from Stewart assessing participant experiences in the military by trained study personnel (30). The outcomes of interest were defined as: demographics, age at time of entering service, service duration, use of a firearm while in service, indoor use of a firearm while in service, estimated total career firearm usage in hours, and occurrence of a TBI with and without loss of consciousness.

The estimate of the total firearm usage over the duration of each participant's military career was calculated using the reported monthly firearm usage estimate and service duration.

### Statistical analysis

All tests of significance were two-tailed and alpha was set as 0.05. Statistical analysis was performed using the program “R,” with the packages “epitools,” “car,” “dplyr,” “stringr,” and “gmodels” (31).

For the outcomes of interest that were continuous we estimated the means and standard deviations for PSP cases and controls, differences in means were assessed using the Student's *t*-test. For outcomes of interest that were binary we report frequencies and proportions.

For binary outcomes, we calculated conditional odds ratios (ORs) relative to the control group. We additionally calculated an adjusted OR for each measure controlling for age and highest level of education. Significance of ORs and adjusted ORs were assessed using the Chi square test.

To control for possible confounding variables after conducting the univariate analysis we then performed a multivariate conditional logistic regression analysis using only the variables with *p* < 0.05 in the univariate analysis.

## Results

### Demographic data

There was a significant difference in the highest level of education between cases and controls, with controls being significantly more likely to report having obtained greater than a high school education compared to cases, as previously reported (5). Similarly, controls reported a significantly greater number of years of schooling when compared to PSP cases. As seen in Table 1, we found no between-group differences in the remaining demographic data, including in military specific demographics such as average age at enrollment in the military and average service duration.

**Table 1:** Demographic results.

	**Cases**	**Controls**	**Significance**
**Number of subjects**	***N*** = **67**	***N*** = **68**	
Age: mean ± SD	71.1 ± 7.5	72.2 ± 7.1	*p* = 0.38
Age at beginning of military service: mean ± SD	20.4 ± 2.6	21.2 ± 3.3	*p* = 0.09
Service duration: mean ± SD	4.9 ± 6.6	5.4 ± 6.7	*p* = 0.63
Marital status at reference year: percent (*N*)			*p* = 0.12
Married	97.0% (65)	89.7% (61)	
Divorced	1.5% (1)	8.8% (6)	
Never married	1.5% (1)	1.5% (1)	
Annual income at reference year: percent (*N*)			*p* = 0.14
Less than $50,000	43.3% (29)	26.5% (18)	
$50,000–$79,999	26.9% (18)	27.9% (19)	
Greater than $80,000	25.4% (17)	41.2% (28)	
Did not report	4.5%(3)	4.4% (3)	
Highest level of education: percent (*N*)			*p* < 0.001
High school education or less	55.2% (37)	23.5% (16)	
Greater than high school education	44.8% (30)	76.5% (52)	
Years of schooling completed: mean ± SD	14.0 ± 2.8	16.2 ± 3.3	*p* < 0.001
Ethnicity: percent (*N*)			*p* = 0.50
White or European-American	98.5% (66)	100.0% (68)	
Black or African American	1.5% (1)	0.0% (0)	
Employment at reference year: percent (*N*)			*p* = 0.40
Full-time employed	79.1% (53)	70.6% (48)	
Part-time employed	1.5% (1)	4.4% (3)	
Retired	19.4% (13)	25.0% (17)	
Dementia screening tools: mean ±*SD*			
Cases–MMSE	27.0 ± 2.1	N/A	
Controls–TICS	N/A	36.5 ± 3.5	

*There was a significant difference between the highest level of education obtained and the years of schooling completed, as previously reported. There were no other between group differences. SD, Standard deviation; MMSE, Mini-Mental Status Exam; TICS, Telephone Interview for Cognitive Status*.

### Firearm usage

We found a significant difference in proportion of participants reporting use of firearms as part of their job in the military between PSP cases (80.6%) and controls (64.7%), yielding an adjusted OR of 1.7 (*p* = 0.04; Table 2). However, there was no significant difference in the proportion of cases and controls who reported indoor firearm use while in the military, OR of 0.7 (*p* = 0.56). We found no significant difference in estimated total hours spent using a firearm in the military between PSP cases (112.9 ± 169.8) and controls (158.6 ± 299.2; *p* = 0.28).

**Table 2:** Firearm exposure results.

	**Cases exposed: percent (*N*)**	**Controls exposed: percent (*N*)**	**OR (95% CI)**	***P*-value**	**Adjusted OR (95% CI)**	***P*-value**
Use of firearm in service	80.6% (54)	64.7% (44)	2.2 (1.0–5.0)	0.04	1.7 (0.7–4.0)	0.04^*^
Indoor use of firearm	7.5% (5)	10.3% (7)	0.7 (0.2–2.4)	0.56	0.7 (0.2–2.5)	0.56

*PSP cases were significantly more likely to report use of firearm while in the service, adjusting for age and education. OR, Odds ratio; N, number*.

* *p ≤ 0.05*.

### Traumatic brain injury

We found that reporting having experienced a TBI was more prevalent in the PSP cases (38.8%) compared to the controls (27.9%). This yielded an OR of 1.6, however, this was not significant (*p* = 0.20; Table 3). However, contrary to what we hypothesized, of those who experienced TBI, loss of consciousness was more prevalent in controls, though this difference was not significant either.

**Table 3:** TBI odds ratios.

	**Cases exposed: percent (*N*)**	**Controls exposed: percent (*N*)**	**OR (95% CI)**	***P*-value**	**Adjusted OR (95% CI)**	***P*-value**
TBI occurrence	38.8% (26)	27.9% (19)	1.6 (0.8–3.4)	0.20	1.8 (0.8–3.9)	0.18
Loss of consciousness in TBI participants	42.3% (11)	57.9%% (11)	0.5 (0.2–1.8)	0.37	0.7 (0.2–2.5)	0.30

*There was a higher prevalence of reported TBI in the cases, but, this was not significant. TBI, traumatic brain injury; OR odds ratio*.

### Multivariate conditional logistic regression

Based on the results of univariate analyses, we included having greater than a high school education and reported use of firearm in military service in the multivariate conditional logistic regression. Concordant with other studies, we found that higher education level is inversely related to the risk of developing PSP, with an OR of 0.3, *p*-value of 0.01. We additionally found that in multivariate analysis reported firearm use during military service remained a significant predictor for developing PSP, yielding an OR of 3.7, *p*-value of 0.01.

## Discussion

In this study we utilized a case-control study design to investigate potential risk factors for developing PSP in a military veteran population. In particular, we focused on firearm usage and reported occurrence of TBI as a risk factors for developing PSP.

We used reported firearm usage as a potential indicator of exposure to heavy metals, based on findings in the literature significant differences in blood lead levels with significant firearm use (13–15). We were interested in investigating heavy metal exposure as a potential risk factor of PSP due to the growing body of literature implicating lead in abnormal brain pathologies, in particular tau pathologies (6–10). Thus, we hypothesized that PSP patients would report greater use of firearms compared to controls. Our hypothesis was supported by the study as we found that cases were more than twice as likely to report use of firearms in their military positions compared to controls. However, we did not find differences in estimated total hours of firearm usage between cases and controls. It is likely that the binary recall of firearm use is more accurate than the estimated hours spent shooting, due to level of detail required for accurate recall. Lead and other metal exposures would be expected to be greatest in indoor firearm use environments. The very small numbers of cases (5) and controls (7) who reported indoor firearm limits interpretation of the findings. We also found, as previously reported, that higher education was associated with PSP (5, 32, 33). More importantly, in multivariate analyses, use of firearms in the military and level of education were both significantly associated with PSP. Moreover, the usage of arms in the military may increase to almost four times the risk of developing PSP, whereas there was an inverse relation with higher education.

This is the first case-control study to demonstrate PSP is associated with exposure to firearm usage. In our main study that included 284 cases and 284 controls matched for age, gender, and race, expert-inferred exposures analysis showed that cases were more often assigned to metal non-military occupational exposures by case-status-blinded experts (5). In addition, expert-inferred exposures analysis showed that PSP patients had greater but not significantly different exposures to manufacturing and mining jobs than controls (not shown). However, it is possible that statistical differences were not found due to the overall low frequency of these jobs in our sample. Moreover, a recent report of a cluster of PSP patients in France in a geographical area with severe environmental contamination by industrial metals supports the possible association with metals (34).

Our study also sought to investigate the role of TBI as a risk factor for PSP. Based on the fact that TBI has been implicated in other tauopathies, including AD and CTE, we hypothesized that PSP patients would be more likely to report head injury than controls. It is hypothesized that TBI may contribute to the development of these conditions by leading to an increase in the amount of aggregated phosphorylated tau (9, 10). Though we did find that a greater proportion of PSP cases reported head injury compared to controls there was no significant difference. However, we did not have enough power in this study to fully assess this relationship, and future studies will be needed. Additionally, participants who endorsed having experienced a head injury were asked if they had loss of consciousness at the time. This was included as a potential marker of head injury severity. Although not statistically significant, contrary to our hypothesis of those who experienced TBI, controls were more likely to report loss of consciousness. Again, however, there was not enough statistical power.

Our study has several limitations. One of the main limitations of our study is that we were unable to test our hypothesis that the connection between firearm usage and the risk of developing PSP was mediated via heavy metal exposure. We based our hypothesis on strong literature support demonstrating that firearm usage increases serum lead content, and that lead exposure acts on the tau pathway increasing gene expression and tau protein levels. It would have been very interesting to be able to test this hypothesis by obtaining serum lead levels on our participants. However, given our study design, along with the significant separation in time from potential exposure to lead and development of clinical PSP symptoms, this was not possible. We hope that additional studies, including potentially prospective studies, will be able to investigate this further. An additional limitation is that we have asked participants to recall information from many years in the past and thus it might not be recalled accurately. We believe many of our primary endpoints, including use of a firearm in their position while in the military and whether they have experienced a head injury are likely to be recalled accurately. However, the more specific endpoints, including estimated amount of time shooting per month, are likely more difficult to recall. There is also the concern that due to the potential effect of PSP on recall would be poorer in the cases. To control for this we included only incident PSP cases and used the MMSE to screen for cognitive impairment. Another limitation of our methodology is that we did not specifically inquire about repetitive head trauma, which is one of the mechanisms thought to contribute to the development of CTE. Focusing more on the nature of head injuries and the number of occurrences would be an interesting direction for future studies. More importantly, it would have been important to obtain the medical records of the study participants while in service to confirm the occurrence and severity of TBIs. This potentially would have allowed for a more accurate estimation of exposure to head injury. Finally, due to the relatively small sample size, our study had limited statistical power.

One of the strengths of this study is that it is the first to investigate these potential risk factors for the development of PSP and it was conducted in a population with higher exposure to these factors than the general population. In addition, the study used a validated questionnaire and uniform telephone administration to cases and controls (30). Additionally, given that PSP is such a rare disease we recruited a relatively large number of cases within the military veteran population.

In summary, our study found that in multivariate analyses, use of firearms in veterans who were in the military increases the odds of PSP, whereas higher education decreases the odds of developing this disorder. Further larger studies should confirm these results and investigate the relationship between PSP and metals, particularly when previous studies show possible association between PSP and metals.

## Ethics statement

This study was carried out in accordance with the Declaration of Helsinki. All subjects provided written informed consent prior entering the study. The protocol was approved by the IRB at each participating site.

## Author contributions

IL, HC, DH, SR, and CC contributed to the conception and design of the study. KK organized the database and performed the statistical analysis, wrote the first draft of the manuscript. IL, HC, DH, SR, and CC critically revised the draft and provided updates. All authors contributed to the manuscript revision, read, and approved the submitted version.

### Conflict of interest statement

The authors declare that the research was conducted in the absence of any commercial or financial relationships that could be construed as a potential conflict of interest.
